# Relation of phosphodiesterase type 5 inhibitors and malignant melanoma: a meta-analysis and systematic review

**DOI:** 10.18632/oncotarget.17518

**Published:** 2017-04-29

**Authors:** Jie Wang, Yigen Shen, Jiaoni Wang, Yangjing Xue, Lianming Liao, Saroj Thapa, Kangting Ji

**Affiliations:** ^1^ Department of Cardiology, The Second Affiliated and Yuying Children's Hospital, Wenzhou Medical University, Wenzhou 325000, Zhejiang, China; ^2^ Department of Oncology, Academy of Integrative Medicine, Fujian University of Traditional Chinese Medicine, Fuzhou 3250112, Fujian, China

**Keywords:** phosphodiesterase type 5 inhibitors, malignant melanoma, basal cell carcinoma, meta-analysis, systematic review

## Abstract

Data on the association between using PDE5 inhibitors and malignant melanoma are conflicting. To estimate the relation of using PDE5 inhibitors with risk of malignant melanoma, Medline (Ovid) and Embase (Ovid) databases were searched up to February 2017, and a random effects model was used to calculate the summary risk estimates. Five observational studies were included. Five studies reports encompassed a total of 15,979 melanoma cases occurring among 1, 188,414 participants. The pooled multivariable-adjusted RR of melanoma in patients with using PDE5 inhibitors was 1.12 (95% CI: 1.03–1.21, I^2^ = 0.48). Findings from this systematic review support that PDE5 inhibitor use is associated with increased risk of melanoma in ED patients, the result remains inclusive and warrants further study in the future.

## INTRODUCTION

Erectile dysfunction (ED) is common and increases as men age. It is estimated to affect over 322 million men worldwide by 2025 [1–3]. Phosphodiesterase type 5 (PDE5) inhibitors, which include sildenafil, tadalafil, vardenafil and avanafil, have been widely prescribed for ED [2]. These drugs enhance the erectile response by inhibiting PDE5, which is responsible for the degradation of cyclic guanosine monophosphate (cGMP) in the cavernous smooth muscles [2]. Animal studies have found that mutations in the BRAF gene result in down regulation of cGMP-specific phosphodiesterase PDE5A, which lead to an increase in colonization of the lungs by melanoma cells [4–6]. So, it has been hypothesized that PDE5 inhibitors used for ED may increase risk of malignant melanoma.

During the recent decade, numerous epidemiologic studies [7–11] have assessed the association between PDE5 inhibitors used to treat ED and the risk of malignant melanoma, and a positive association (i.e., RR >1.00) was reported in three studies. But the original studies on this issue have doubled. Therefore, we conducted a meta-analysis to investigate the relation of using PDE5 inhibitors with risk of malignant melanoma.

## RESULTS

### Literature search

**Figure 1:**
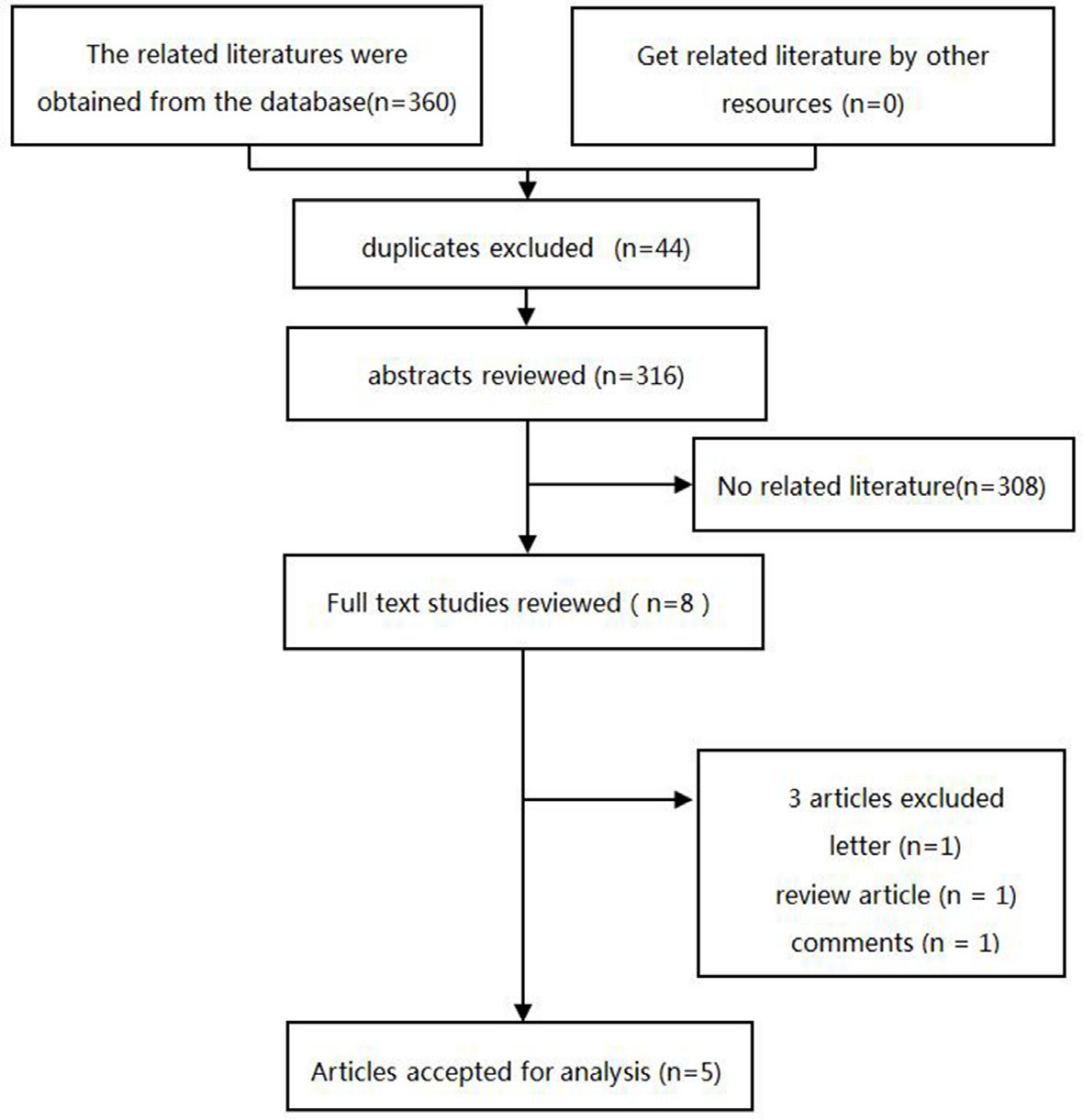
Flowchart of the meta-analysis of phosphodiesterase type 5 inhibitors and risk of malignant melanoma

The initial search strategy found 360 citations. Of these, we included 8 articles after review of the title and abstract. After detailed examination, 3 literatures were excluded (reasons shown in Figure 1). In total, 5 articles were included in our meta-analysis. A flow chart showing the study selection is presented in Figure 1.

### Study characteristics

Table 1 presents characteristic of the 5 studies [7–10] in the meta-analysis. The studies were published in the past 2 years. Two studies was conducted in North America, and the rest in Europe. There were one nested case-control study, one parallel case–control studies and three prospective cohort studies. Overall, five studies reports encompassed a total of 15,979 melanoma cases occurring among 1,188,414 participants. The sizes of cases diagnosed melanoma ranged from 144 to 7,045 (total15,979). The sizes of participants ranged from 77,495 to 706,037 (total1, 188,414). Only two studies [9, 10] reported the mean follow-up years (4.9 years for both, Table 1).

**Table 1:** Characteristics of observational studies of phosphodiesterase type 5 inhibitors and risk of malignant melanoma included in this meta-analysis

Study	Regions	Design	No. of cases/no. of participants	Study period	Meanfollow-up(y)	Adjustment	Adjusted RR(95% CI)	Quality score
Matthews et al. 2016[10]	UK	Matched cohort	1,315/706,037	1999-2014	4.9	Age, BMI, Smoking, alcohol use	1.14 (1.01–1.29)	Selection: 4Comparability:2Outcome:2
Loeb et al. 2015 [9]	Swedish	Case-control	4,065/24,390	2006-2012	NR	Educational level, CCIDisposable income marital status.	1.21(1.08,1.36)	Selection: 3Comparability:2Exposure:2
Li et al.2014[7]	US	Cohort	142/204,870	2000-2010	NR	Age, BMI, smoking,physical activity,childhood reaction to sun, number of sunburns, hair color, mole count, family history of melanoma,sun exposure, UV index, other treatment for ED.	1.84(1.04,3.22)	Selection: 4Comparability: 2Outcome:3
Lian et al. 2016[8]	UK	Cohort	440/143,343	1998-2014	4.9	Age, BMI, year of cohort entry, smoking, alcohol-related disorders, precancerous skin lesions, presence of naevi, number of different drug classes used, health-seeking–related variables immunosuppression,use of antiparkinsonian drugs, Charlson comorbidity scorenumber of physician visits in the year before cohort entry.	1.18(0.95,1.47)	Selection:4Comparability: 2Outcome:2
Pottegard et al. 2016[11]	DNHR(Denmark)	Casecontrol	7045/77495	2000-2012	NR	Use of oral steroids, weak/moderate topical steroids, strong/very strong topical steroids, thiazides, beta-blockers, angiotensinII, receptor blockers, low-dose aspirin (only in the DNHR), non-aspirin non-steroidal anti-inflammatory drugs, antidepressants, and statins; diagnoses of non-melanoma skin cancer, diabetes, chronic obstructive pulmonary disease, alcohol-related disease, and moderate to severe renal disease; and highest education achieved (in the DNHR) and socioeconomic level based on the US Census block of residence (in the KPNC database).	1.06 (0.96–1.18)	Selection: 3Comparability: 2Exposure:2
Pottegard et al. 2016[11]	KPNC(US)	Casecontrol	2972/32279	2000-2012	NR		1.01 (0.91–1.12)	Selection: 3Comparability: 2Exposure:2

BMI: body mass index; CCI: Charlson comorbidity index; NR: no reference; DNHR: Danish Nationwide Health Registries; KPNC: Kaiser Permanente Northern California.

Also shown in Table 1 are the relative risk calculated for each individual report included in the pooled analysis, along with its 95% confidence interval. All studies had a relative risk greater than 1.0. Three studies reported a statistically significant association between the use of PDE5 inhibitors and melanoma incidence. Four studies are adjusted for a wide range of potential confounders, including age, body mass index, smoking and alcohol use.

### Main analysis

**Figure 2:**
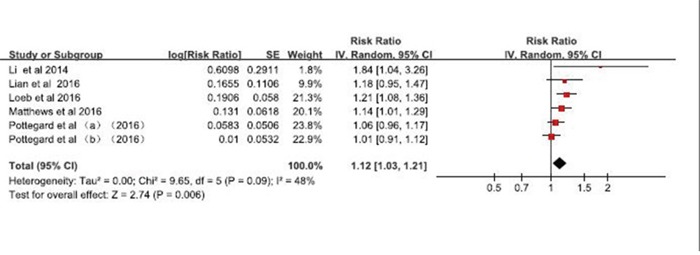
Forest plot of studies examining the association between phosphodiesterase type 5 inhibitors and risk of malignant melanoma

Five studies (one nested case-control study, one parallel case–control studies and three prospective cohort studies) were included in the meta-analysis. Three studies reported a positive association (i.e., RR >1.00), and two studies reported RR >1.00 but not statistically significant. Moderate heterogeneity was detected (P = 0.09, I^2^=0.48), and the multivariable-adjusted RR (95% CI) from the random-effects model was 1.12 (1.03-1.21; Figure 2).

**Figure 3:**
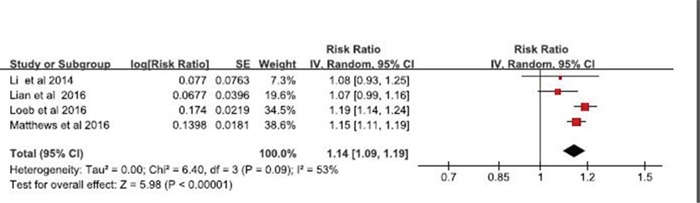
Forest plot of studies examining the association between phosphodiesterase type 5 inhibitors and risk of basal cell carcinoma

Four studies also reported data on risk associated between the use of PDE5 inhibitor and basal cell carcinoma. Interestingly, a summary relative risk derive from multivariable-adjusted RR was 1.14 (95% CI, 1.09-1.19), with moderate evidence of heterogeneity (p=0.09, I^2^=0.53) (Figure 3).

### Subgroup and sensitivity analyses

Subgroup analyses were conducted to explore potential sources of heterogeneity in the association between using PDE5 inhibitors and malignant melanoma. Table 2 presents results of subgroup analyses of malignant melanoma incidence according to study regions, case numbers, types of drugs, study quality, study design and adjustment for sun exposure. The association between PDE5 inhibitors intake and melanoma risk was statistically significant in sildenafil users (RR:1.28, [95% CI, 1.06-1.21]), whereas different effect was found in vardenafil or tadalafil users (RR:1.28, [95% CI, 0.93-1.76]). There was a statistically significant association among cohort studies(RR:1.18, [95% CI, 1.03-1.36]), but not among case–control studies (RR:1.09, [95% CI, 0.98-1.20]. And the result of subgroup analyses according to study quality got similar result. We also found statistically significant association among studies including ≥500 cases (RR:1.10, [95% CI, 1.02-1.19]), as well as those conducted in Europe (RR:1.13, [95% CI, 1.06-1.21]).

**Table 2:** Stratified analyses of phosphodiesterase type 5 inhibitors associated with malignant melanoma

	RR (95% CI)	No. of reports	I^2^(%)	P_Heterogeneity_
Study regions				
US	1.27(0.72,2.26)	2	76	0.04
Europe	1.13(1.06,1.21)	4	5	0.37
Study design				
Cohort	1.18(1.03,1.36)	3	23	0.27
Case–control	1.09(0.98,1.20)	3	64	0.06
Study quality				
High (8-9)	1.18(1.03,1.36)	3	23	0.27
Low (≤7)	1.09(0.98,1.20)	3	64	0.06
Case numbers				
≥500	1.10(1.02,1.19)	4	51	0.11
<500	1.36(0.91,2.04)	2	51	0.15
Types of drugs				
Sildenafil	1.28(1.14,1.44)	5	32	0.20
Vardenafil or tadalafil	1.28(0.93,1.76)	4	70	0.04
Adjustment for sun exposure				
Yes	1.84(1.04,3.26)	1	NA	NA
No	1.10(1.03,1.18)	5	39	0.16

We performed a sensitivity analysis to investigate the influence of a single study on the overall risk estimate by omitting each single report from the meta-analysis. The results showed the overall risk estimates did not substantially influenced by any single study, with a range from 1.09 (95% CI: 1.00–1.18) to 1.15 (95% CI: 1.06–1.24) for risk of malignant melanoma, which implied that our results were statistically reliable.

## DISCUSSION

In this meta-analysis of 5 population-based observational studies, our findings show that the risk of malignant melanoma is increased by 12% for those who were ever users of PDE5 inhibitor for ED.

The underlying biological mechanisms involved in the association between PDE5 inhibitors and malignant melanoma are not clear. The RAS/RAF/MEK/ERK pathway plays a key role in melanoma cell proliferation and survival [6] It was previously observed that the high cGMP levels in response to CNP appeared to correlate with the aggressiveness/invasiveness of the tumor cells [12]. Arozarena et al. [4] found that oncogenic *BRAF* mutation promotes the invasion of melanoma cells by down-regulating PDE5A and elevate cGMP levels through the MEK and the transcription factor BRN2. They discovered that PDE5A promote melanoma cell invasion through cGMP, Ca2+, and increased contractility.

Moreover, Dhayade et al. [5] recently uncovered a previously unknown cGMP-cGKI signaling cascade in murine and human melanoma cells. They document a cGMP-dependent growth-promoting pathway, of which activation promotes melanoma cell growth and migration in a p44/42 MAPK-dependent manner, both in murine and human melanoma cells.

Interestingly, analysis on Basal Cell Carcinoma also shows that the risk of malignant melanoma is increased by 14% of the user of PDE5 inhibitor, though there is no clear biological mechanism for a possible association between PDE5 inhibitor use and basal cell carcinoma. Thus, the potential use of PDE5 inhibitor for melanoma incidence deserves further investigation.

Several limitations should be acknowledged as well. First, this is a meta-analysis of population-based observational studies so we can demonstrate the association but not a causal relationship. We cannot draw a conclusion that PDE5 inhibitor itself or other unmeasured or uncontrolled confounders, especially sun exposure, are the cause of the increased malignant melanoma, because weakness inherent in observational studies is that they may be subjected to confounding. Second, assessments of ED differ in each study, which included diagnosis from other studies [8, 9], national databases [10] and self-reported data [7]. It is likely to induce the population selective bias. Third, we did not conduct dose-response meta-analysis., because of insufficient data of drug dose.

A major strength of our study is that data were from good quality observational studies. Besides, each study included a large number of patients and was followed up long enough for outcomes to occur. Moreover, diagnoses of melanoma are identified clinical database in 3 studies [8–10]. In the fourth study [7], the diagnosis was confirmed by physicians. In addition, we conducted sensitivity analyses to assess the robustness of our findings, which produced generally consistent results.

We hope that the results of the present analysis will contribute to the design of future studies addressing the tissue. Studies that evaluate the association of dose and frequency of PDE5 inhibitors and melanoma risk are needed. Furthermore, more known skin cancer risk factors, including alcohol-related disorders, smoking status, body mass index, the presence of naevi, precancerous skin lesions, use of antiparkinsonian drugs, and immunosuppressants need to be considered in the study.

In conclusion, this meta-analysis provides evidence that PDE5 inhibitor use is associated with increased risk of melanoma in ED patients, the result remains inclusive and warrants further study in the future.

## MATERIALS AND METHODS

This meta-analysis was performed according to the guidelines of the Meta-analysis of Observational Studies in Epidemiology group (MOOSE) [13].

### Search strategy

We first searched the literature in any language in April 2015 of the Medline (Ovid) and EMBASE (Ovid) using the following search terms ” PDE5-Is”,” phosphodiesterase type 5 inhibitors”, ”sildenafil”, “tadalafil”, “vardenafil”,” malignant melanoma”,” melanoma” and ” Skin Neoplasms”. To make sure our study was based on up-to-date results, we further updated the literature search of Medline (Ovid) and EMBASE (Ovid) on February 20, 2017. Additional studies were identified through the reference lists of relevant reports and relevant reviews.

### Study selection

Two investigators (J.W and Y.G.S) independently evaluated the titles or abstracts, or both, of the selected reports and assessed the full-text articles for eligibility. Any uncertainty regarding eligibility was resolved by discussion, or by consulting with the third investigator (K.T,J.). Studies were eligible for our analysis if: (1) the exposure of interest was PDE5 inhibitors; (2) the outcome of interest was malignant melanoma; (3) the study was a observational study (i.e., case–control or cohort study); and (4) were original studies published in peer-reviewed journals (i.e., not review articles, comments or conference abstracts). A study must meet all the four inclusion criteria for inclusion. In the case of multiple publications, we chose the articles with the largest sample or the longest follow-up interval. Studies reporting crude associations without any adjustment were also excluded.

The agreement between the 2 investigators was 99.1% for the first screen and 100% for the full-text articles.

### Data collection

We extracted the following information using a standardized, pre-defined data extraction form: name of first author; publication year; study location; number of participants; number of cases; mean baseline age; study period and mean follow-up years; adjustment covariate and effect size; and quality score. If the data was unavailable, we corresponded with the author(s) for the relevant data.

### Assessment of quality

Studies may differ in quality, a subjective assessment of methodological quality for nonrandomized studies was evaluated by using the Newcastle–Ottawa Scale (NOS) [14]. The NOS is a tool to assess the quality of nonrandomized studies, which is endorsed by the Cochrane Collaboration in its 2011 handbook [14]. It used a star system based on three perspectives: the selection of the study groups, the comparability of the groups, and the assessment of outcome or exposure [15]. A total score of 8–9 was deemed high quality [15].

### Data synthesis and analysis

The relative risk (RR) was used as the common measure of association of using PDE5 inhibitors and malignant melanoma, and the hazard ratio (HR) or odds ratio (OR) was considered equivalent to the RR [16], while the OR was converted to RR by the formula RR=OR/[(1-Po)+(Po×OR)], in which Po is the incidence of the outcome of interest in the nonexposed group [17]. Forest plots were produced to visually assess the RR and corresponding 95% confidence interval (CI) across studies. The presence of heterogeneity across studies was evaluated by the Q statistic (significance level: p<0.10) and the I^2^ statistic (ranges from 0% to 100% with lower values representing less heterogeneity) [18]. The RR was pooled using the DerSimonian and Laird inverse-variance-weighted random-effects models [12].

We conducted pre-specified subgroup analyses to examine the impacts of various study characteristics, including regions, case numbers, types of drugs, number of prescriptions, study quality, study design and adjustment for Sun exposure. Sensitivity analysis was performed to assess the influence of individual study on the summary risk estimate by omitting one study in each turn and then reanalyzing the remaining ones.

Analyses were performed with the Review Manager software (version5.2 for Windows; the Nordic Cochrane Centre, Copenhagen, Denmark). All statistical tests were 2-sided and α<0.05 was considered statistically significant.

## Abbreviations

CIs: confidence intervals
RR: relative risk
HR: hazard ratio
PDE5 inhibitors: Phosphodiesterase type 5 inhibitors
ED: Erectile dysfunction
cGMP: cyclic guanosine monophosphate
NOS: Newcastle–Ottawa Scale.
